# Correlation between Phylogroups and Intracellular Proteomes of *Propionibacterium acnes* and Differences in the Protein Expression Profiles between Anaerobically and Aerobically Grown Cells

**DOI:** 10.1155/2013/151797

**Published:** 2013-06-26

**Authors:** Itaru Dekio, Renata Culak, Min Fang, Graham Ball, Saheer Gharbia, Haroun N. Shah

**Affiliations:** ^1^Department for Bioanalysis and Horizon Technologies, Microbiology Services, Health Protection Agency Colindale, London NW9 5EQ, UK; ^2^School of Science and Technology, Nottingham Trent University, Nottingham NG11 8GS, UK

## Abstract

*Propionibacterium acnes* is one of the dominant commensals on the human skin and also an opportunistic pathogen in relation to acne, sarcoidosis, prostate cancer, and various infections. Recent investigations using housekeeping and virulence genes have revealed that the species consists of three major evolutionary clades (types I, II, and III). In order to investigate protein expression differences between these phylogroups, proteomic profiles of 21 strains of *P. acnes* were investigated. The proteins extracted from cells cultured under anaerobic and aerobic conditions were analysed using a SELDI-TOF mass spectrometer, high-resolution capillary gel electrophoresis, and LC-MS/ MS. The SELDI spectral profiles were visualised as a heat map and a dendrogram, which resulted in four proteomic groups. Strains belonging to type I were represented in the proteome Group A, while Group B contained type III strains. Groups C and D contained mixtures of types I and II. Each of these groups was not influenced by differences in culture conditions. Under anoxic growth conditions, a type IB strain yielded high expressions of some proteins, such as methylmalonyl-CoA epimerase and the Christie-Atkins-Munch-Petersen (CAMP) factor. The present study revealed good congruence between genomic and proteomic data suggesting that the microenvironment of each subtype may influence protein expression.

## 1. Introduction

*Propionibacterium acnes* is one of the most dominant commensals of the human skin (see, e.g., Dekio et al. [1]; Grice et al. [2]). It is a facultative anaerobe habiting the hair follicles to avoid the well-aerated surface of the skin [3]. *P. acnes* has long been considered as a pathogen of acne vulgaris (common acne) although this is still not proven conclusively [4]. However, despite its association with the skin, *P. acnes* is increasingly reported in association with various infections and conditions, including prosthetic joint infection [5], prostate cancer [6], and sarcoidosis [7].

Three major genetic divisions, known as types I, II, and III, are currently recognised, and it has been shown that both types I and II share classical coryneform cell morphologies of coryneform rods, while type III has the capacity to be filamentous [8]. All of these types can be isolated from normal human skin, but only type IA is considered as the “acne-specific” subtype [9, 10]. Further subtyping of the type I division into types IA_1_, IA_2_, IB, and IC by multilocus sequence typing (MLST) based on housekeeping and virulence genes has now also been described [11, 12]. We have recently shown by MLST analysis that strains isolated in Japan belong to five of these six phylogroups (IA_1_, IA_2_, IB, II, and III) which exist both in the Western and the Eastern countries [13]. 

In order to investigate the possible association of these subtypes with disease, the biochemical profiles of various phylotypes have been investigated by other groups, but so far the underlying basis for their distribution remains elusive. In order to perform more in-depth analysis, we conducted a detailed proteomic analysis of our *P. acnes* strains, previously typed by MLST [13] and grown under different culture conditions.

The ability of *P. acnes *to grow in air, under microaerophilic and anaerobic conditions, is likely to contribute to the success of the organism to transfer from its normal habitat of the skin to the anoxic environment of deeper seated systemic infections. Such a change requires the expression of different factors for the organism to adapt to its new environment, while also shifting its metabolism towards anaerobic growth. Consequently, one of the aims of the present study was to assess whether it is possible to follow the transitional changes of the proteome under these growth conditions in a selected strain.

## 2. Material and Method

### 2.1. Isolation and Characterisation of *P. acnes* Strains

Twenty-one *P. acnes* strains were collected from the skin surface of healthy volunteers and patients with atopic dermatitis in Japan. These were a part of a collection of fifty strains used in a recent study by Dekio et al. [13] in which the genetic diversity of *P. acnes* strains was reported against the genetic diversity and distribution of *P. acnes* strains from Japan and Europe using MLST. The isolation procedure was performed as described previously, using a moistened swab and anaerobic culture technique with minor modifications [1, 13]. In brief, the open end of a sterile plastic cylinder was manually placed on the sampling site and the inside area was scrubbed by using a PBS-moistened swab. Then the tip of the swab was broken into a glass tube containing PBS. The liquid sample is serially diluted from 10^−1^ to 10^−5^ and plated out for anaerobic culture. 

The strains were identified to species level using 16S rDNA gene sequencing and further typed by analysing seven housekeeping genes (*aroE*, *atpD*, *gmk*, *guaA*, *lepA*, *recA*, and *sodA*) according to the original MLST scheme of McDowell et al. [11], which is accessible online at the *Propionibacterium acnes* MLST Database website, http://pubmlst.org/pacnes/. The profiles of these strains are shown in Table 1. 

### 2.2. Extraction of Proteins from *P. acnes* Strains

The strains were plated on Columbia Blood Agar (CBA) plates and incubated at 37°C under anaerobic condition (85% N_2_, 10% H_2_, 5% CO_2_, <0.02% O_2_ in Whitley A35 Anaerobic Workstation, Don Whitley Scientific, Shipley, UK), while aerobic growth utilised a standard incubator. In addition, strain K115 was grown under microaerophilic conditions by using CampyGen gas generating system (Oxoid) with an anaerobic jar to examine the proteome of the strain, which produced luxuriant growth under aerobic, anaerobic, and microaerophilic condition. We have previously examined the stability of SELDI-TOF-MS profiles of cells cultured in liquid media at mid-log and stationary phases against those grown on agar plates and have consistently found the latter to produce mass spectral profiles that are more reproducible and therefore more useful for strain characterisation [14]. For the present study, cells were therefore plated out on CBA. Strains varied in their growth rates, some showing luxuriant growth after 2 days whereas others took up to 5 days to reach the same level, both in anaerobic and aerobic culture conditions. All cultures were therefore incubated for 5 days for both conditions when they would have reached stationary phase growth. 

The proteome profiles of several strains of types IA_2_, IB, II, and III were analysed up to 7 days to ensure that cells had reached the stationary phase. However, the profiles of cells grown 5 and 7 days were almost identical. We selected day 5 for all further analyses because it was easier to harvest cells up to this point as the medium was still intact. Beyond day 5 the medium began to dry out and we anticipate that growth pellet would contain more dead cells.

After 5 days of incubation, protein samples were extracted by a standard lysis method using 8 M urea, 2% CHAPS, and 40 mM Tris [14]. The cells were harvested into 500 *μ*L of the standard lysis buffer containing 300 mg of glass beads. The samples were placed in a FastPrep device (MP Biologicals) and disrupted for a total of 60 seconds (3 × 20 sec with 5 min cooling between them). The samples were then centrifuged at 21 000 ×g for 30 min and the supernatants were collected. Protein concentration of the extracts was measured using the Bradford assay [15].

### 2.3. SELDI-TOF Mass Spectrometry

Q10 (Weak Cation Exchanger ProteinChip) was used for this analysis. The wells of the ProteinChip arrays were preactivated for 15 min with 5 *μ*L of 20 mM Tris/HCl pH7.4 containing 0.1% Triton X-100. The buffer was decanted and 6 *μ*g of total protein in preactivation buffer was applied onto each spot. The ProteinChip was left in a humidity chamber for 1 h. After the incubation, the unbound sample and the buffers were removed and the spots were washed three times with 20 mM Tris/HCl pH 7.4 containing 0.1% Triton X-100. The chip was dried and 0.5 *μ*L of sinapinic acid was applied twice onto each spot. The ProteinChip arrays were analysed in a MALDI-TOF mass spectrometer (Ciphergen BioSystems, Model PBS II) according to an automated data collection protocol [14]. The spectra were generated at laser intensity of 230, high mass of 50 kDa, detector sensitivity of 10, and focus mass of 25 kDa. The instrument was operated in positive ion mode and a nitrogen laser emitting at 337 nm was used. All mass ions from each spectrum were analysed using the Ciphergen Biosystems Express Heat Map Software as described previously [14]. This software allowed comparison of mass ions between various samples and presents the data both as a dendrogram and Heat map in which coloured boxes are used to visualise the expression of proteins. 

The analysis to identify the peaks that differ between anaerobic and aerobic cultures was done by stepwise calculation, by creating mean spectra of anaerobic culture samples and aerobic culture samples of all strains. This approach was based on utilisation of an artificial neural network approach outlined in Matharoo-Ball et al. [16]. The ANN approach encompassed supervised training using a multilayer perceptron model employing two hidden nodes with a sigmoidal transfer function. The samples were subjected to the Monte Carlo Cross Validation strategy by randomly segregating them into three different subsets, namely, train (to enable learning), test (for early stopping when the network fails to perform better with a threshold of 3000 epochs or 1000 epochs without improvement in mean square errors (MSE)), and validation subsets (to authenticate the model performance on previously unseen data) in proportions of 60%, 20%, and 20%, respectively. Each ion from the mass spectrometry was used as individual input variable to the model. The performance of the model was used to rank the predictive capability of the ions and thus determine the most important and select them for further analysis.

### 2.4. High-Resolution Capillary Gel Electrophoresis (CGE)

To ascertain if gross changes in protein expression would be visible between cells grown aerobically and anaerobically, protein extracts were resolved further using a new, rapid high resolution protein separation technology based upon capillary electrophoresis (deltaDOT) and conventional SDS-PAGE (see Figure 3). Because strain K72 (type IA_2_) showed haemolysis under anaerobic growth but not in aerobic growth, it was selected to assess the potential of the method. Samples (1.3–2.7 mg/mL) were desalted using Pierce desalting columns prior to analysis. Samples were run using a high-resolution capillary gel electrophoresis (CGE) protocols (deltaDOT, UK) under reducing conditions. CGE is a method by which SDS-coated proteins move through the sieving matrix and are separated according to their size. Data was analysed using both deltaDOT's Equiphase Vertexing Algorithm (EVA) and Generalised Separation Transform (GST) algorithm [17, 18].

### 2.5. One-Dimensional Gel Electrophoresis and Protein In-Gel Tryptic Digestion

Protein extracts were separated by one-dimensional SDS-PAGE (1D-SDS-PAGE) using NuPAGE Novex 4–12% Bis-Tris gels (1.0 mm, 12 well, Invitrogen, UK). Ten *μ*g of protein extract was loaded and separated using MOPS running buffer (Invitrogen, UK), in accordance with the manufacturer's instructions. Protein bands were visualised by staining with Coomassie Brilliant Blue G (Sigma-Aldrich, UK). Selected stained protein bands were excised from gel and subjected to in-gel tryptic digestion following the well-established procedure employed by authors' laboratory [19]. Resulting tryptic peptide mixtures were stored at −80°C for further analysis.

#### 2.5.1. Liquid Chromatography and Mass Spectrometry (LC-MS/MS) Using Orbitrap Mass Spectrometer

We selected a strain K115 (type IB), that grew well under aerobic, anaerobic, and microaerophilic conditions and used LC-MS/MS to identify some of the major differentially expressed proteins. We assumed that this strain has largely different proteomic profiles in different culture conditions, because it showed haemolysis under anaerobic culture condition but not under aerobic. The proteolytic digests of the protein extracts from 1 cm segments of the SDS-PAGE gel were further separated by an Ultimate 3000 nano/capillary HPLC system (Dionex, UK) and analysed by a Thermo LTQ Orbitrap mass spectrometer (Thermo Electron, Bremen, Germany) equipped with a nanospray ionization source. Peptide mixtures were initially trapped and desalted in a reversed-phase trap column (C18, 300 *μ*m i.d. × 3 mm, Dionex Ltd., UK) and further separated on a analytical reversed-phase (RP) nanocolumn (C18, 3 *μ*m particle size, 75 *μ*m i.d. × 15 cm, Dionex Ltd., UK). Separation was achieved using a 45-minute linear gradient of 10 to 45% solvent B (90% CH_3_CN/0.1% formic acid) versus solvent A (2% CH_3_CN/0.1% formic acid), then to 90% B for an additional 5 mins, at a flow rate of 300 nl/min.

The mass spectrometer was operated in positive mode with spray voltage at 1.6 kV, capillary voltage at 38 V, capillary temperature at 200°C, and tube lens at 125 V. Helium was used as collision gas but no sheath and auxiliary gas were applied. Tandem MS (MS/MS) data was acquired in data-dependent mode with automatic switching between MS and MS/MS modes. A normalised collision energy of 35%, an activation of *q* = 0.25, and activation time of 30 msec were applied in MS/MS acquisition. The precursor ion scan (*m/z* 440–2000) was acquired in the Orbitrap with a resolution *R* = 60000 at *m/z *400. The six most abundant peptide precursor ions detected in the preceding survey scan were dynamically selected and subjected for collision-induced dissociation (CID) in the linear ion trap to generate MS/MS spectra. The lock mass option, using the polydimethylcyclosiloxane ion generated in the electrospray process from ambient air, the protonated (Si(CH_3_)_2_O)_6_ at *m/z *445.120025, was used for internal recalibration in real time to enable accurate mass measurement. Samples were analysed in a format of technical triplicates. 

The raw MS/MS data files were searched against a nonredundant NCBI database using Mascot v. 2.2.2 search engine (Matrix Science Ltd., UK). The search parameters applied in the database searches were enzyme: trypsin; fixed (or static) modifications: carbamidomethylation of cysteines; variable modifications: oxidation of methionine; missed cleavage sites: 2; precursor mass tolerance ±10 ppm; fragment mass tolerance ±0.8 Da.

## 3. Results

### 3.1. SELDI-TOF Mass Spectrometry

**Figure 1:**
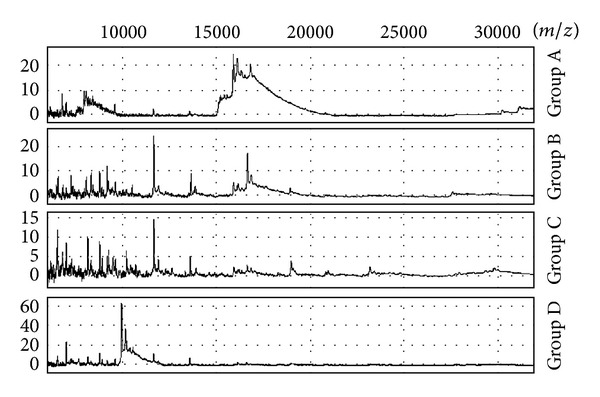
Examples of typical SELDI-TOF MS spectra obtained from the lysates of anaerobically and aerobically cultured cells.

SELDI-TOF mass spectrometry revealed four distinct mass spectral groups in the mass range 10 kDa–35 kDa, with each group (designated as Groups A–D) showing distinctive spectra (Figure 1). The SELDI mass spectral profiles are visualised in Figure 3 which is based on a Heat map and a dendrogram. At a similarity level of 50%, three proteome groups were derived, designated A, B, and C. Interestingly, all strains that belonged to type I were recovered in the proteome Group A, while Group B contained type III strains. Group C contained a mixture of types I and II. By increasing the similarity level to 60%, Groups A and B remained stable whereas Group C resolved into two groups which were designated C and D. Group C strains comprised 4 type I and 7 type II proteomes, while Group D strains contained mostly type II (11 strains) and 2 type I proteomes. Irrespective of growth conditions (i.e., aerobic or anaerobic) the proteome groups remain stable for the majority of the strains; the exceptions were four strains of type I (K51, K72, K81, and K86) which shifted from proteome Group A to C and K15 (type II) which moved from proteome Group D to C. 

**Figure 2:**
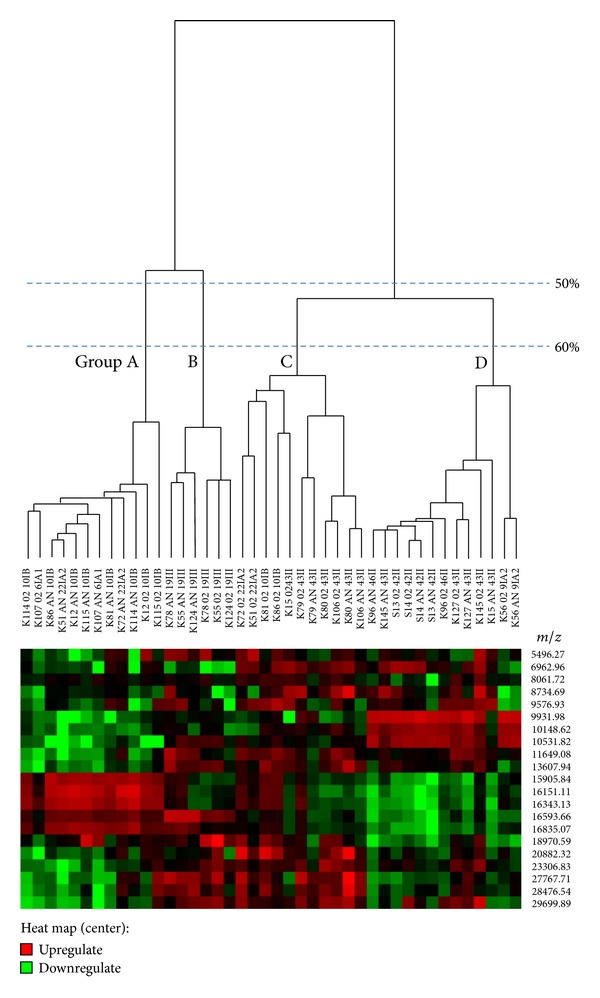
Heat map of SELDI-TOF MS spectra generated from 42 protein samples (anaerobically and aerobically grown cells of 21 *P. acnes* strains). All mass ions from each spectrum were analysed using the Ciphergen Biosystems Express Heat Map Software [14]. This software allowed comparison of mass ions (*m/z*) between various samples and presents the data both as a dendrogram and heat map. The coloured boxes represent intensity of each peak in the SELDI spectra and each colour intensity of the green and the red indicates the difference from the average peak intensity.

Group A profiles were present in nine out of ten strains with types IA_1_, IA_2_, and IB. Group B consisted of four out of nine strains of type II. The three Group C strains belonged to type III. The mass spectra of Group D strains included five other strains within type II, with one atypical strain belonging to type IA_2_ (K56). All groups showed specific differences in mass spectral profiles between anaerobically and aerobically grown cells. The distinct features of these spectral groups were visualised as a heat map (see Figure 2).

To identify peaks which were significantly different between anaerobic and aerobic condition samples, a stepwise ANN modelling approach was conducted to identify an optimised panel of ions that was classified between anaerobic and aerobic; this process identified three ion peaks (Table 2). Among these, a peak at data point 13192 was characteristic to anaerobic culture condition, and other two peaks at data points 9493 and 19134 were to aerobic culture condition.

Strain K115 produced luxuriant growth under aerobic, microaerophilic, and anaerobic conditions. Because of this, this strain was specifically grown under microaerophilic conditions to see if the reduced oxygen would be manifested in a gradual change of the proteome from aerobic to anoxic conditions. The results showed that as soon as the oxygen supply is reduced to microaerophilic level, the cell switches to anaerobic metabolism (data not shown).

### 3.2. High-Resolution Capillary Gel Electrophoresis (CGE)

**Figure 3:**
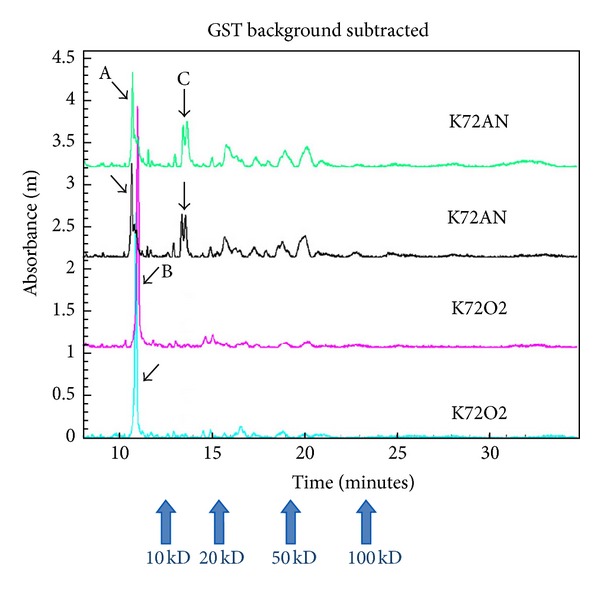
High-resolution capillary gel electrophoresis (CGE) spectra of strain K72 (type IA_2_). The analysis was done in duplicate for each of anaerobic and aerobic culture samples. K72AN: anaerobic culture, K72O2: aerobic culture. Peaks designated A, B, and C were differentially expressed between aerobic and anaerobic metabolism. Proteins designated A and C were visibly downregulated during aerobic growth whereas proteins labelled B were significantly upregulated.

Comparative protein analysis of two lysates from strain K72 (type IA_2_) revealed two large peaks (designated as A and B) in the anaerobic culture sample whereas a more depressed peak was present in the aerobic culture sample. Peak A is located at <10 kDa protein which was expressed maximally in the anaerobic sample but absent in the aerobic lysate. Similarly, peak B consisted of two protein peaks at 15.6 kDa and 16.2 kDa which were present in the anaerobic sample only (Figure 3). Peak B correlated with the mass ion in the SELDI-TOF spectra of Group A, but it was not possible to verify this as both are based solely on pattern matching. 

### 3.3. LC-MS/MS Using Orbitrap Mass Spectrometer Combined with Gel Analysis

**Figure 4:**
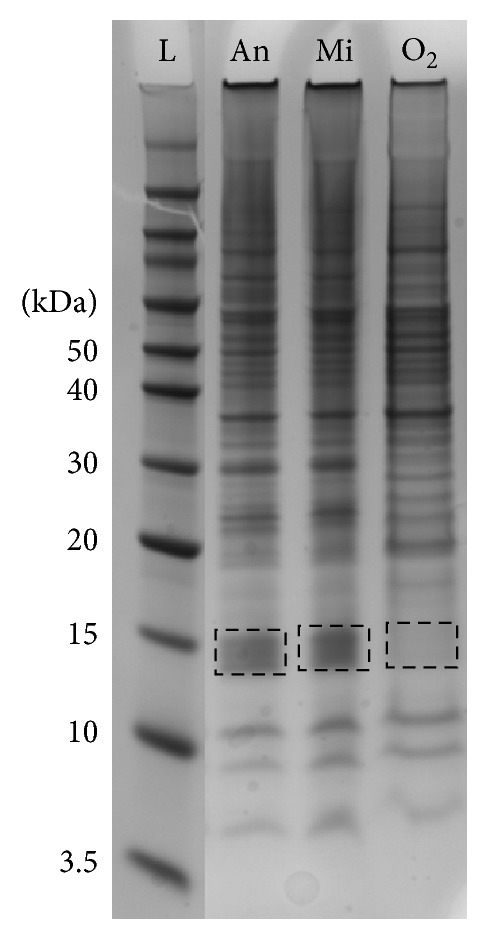
SDS-PAGE band patterns of three different culture conditions in strain K115 (type IB). L: ladder, An: anaerobic culture, Mi: microaerophilic culture, O_2_: aerobic culture. SDS-PAGE analysis of the three extracts indicated a high degree of similarity between the anaerobic (An) and microaerophilic (Mi) lysates but substantial differences between these (An and Mi) and the aerobic extract (O_2_). In particular, the 12–15 kDa segment of the gel showed a marked difference between both, with negligible proteins visible in the aerobic extract. To gain insight into the proteins that may be involved in this change to anaerobic growth, this segment of the gel was removed, digested with trypsin, and subjected to LC-MS/MS as described in the Materials and Methods section.

Because strain K115 (type IB) has the capacity to grow under anaerobic, microaerophilic, and aerobic conditions, we conducted LC-MS/MS analysis under the three different growth conditions. 1D-SDS-PAGE gel band patterns (Figure 4) showed that anaerobic and microaerophilic cultures resulted in very similar protein expression profiles whereas in the aerobic culture sample some of the major bands were not visible. In particular, we observed a significant increase in protein expression for anaerobically and microaerophilically grown cells at around 12–15 kDa (arrows in Figure 4). For further analysis, this gel range between 12 and 15 kDa was excised from the anaerobic lysate lane of the 1D gel, proteolytically digested, and subjected to nanoLC-MS/MS analysis. Nineteen proteins were identified as a result (according to the following list of proteins). These include housekeeping proteins, such as DNA-binding protein HU; phosphotransferase system, phosphoenolpyruvate-dependent sugar EIIA 2; thiol specific antioxidant, a member of AhpC/TSA family; methylmalonyl-CoA epimerase, an enzyme involved in the propionyl-CoA metabolism; C4-type zinc finger protein, a zinc finger-containing member of the DksA/TraR family; nucleic acid-binding domain protein; osmotically inducible protein C (OsmC-like protein), a stress-induced protein; endoribonuclease L-PSP, an enzyme involved in the aromatic amino acid biosynthetic pathway; DoxX protein, a protein with unknown function; ribosomal proteins such as ribosomal proteins L19, L20, L21, L24, and S10; and a member of the CAMP factor protein family.

List of proteins identified from the gel range 12–15 kDa where proteins were upregulated in strain K115 (type IB) cultured under anaerobic condition is as follows.

 Protein name
   Conserved hypothetical protein
   CAMP factor
   DNA-binding protein HU
   Phosphoenolpyruvate-dependent sugar phosphotransferase system, EIIA 2
   Antioxidant, AhpC/TSA family
   Methylmalonyl-CoA epimerase
   C4-type zinc finger protein, DksA/TraR family
   Nucleic acid-binding domain protein
   OsmC-like protein
   Endoribonuclease L-PSP
   DoxX protein
   30S ribosomal protein S13
   50S ribosomal protein L7/L12
   Ribosome-binding factor A
   Ribosomal protein L19
   Ribosomal protein L20
   Ribosomal protein L21
   Ribosomal protein L24
   Ribosomal protein S10.

## 4. Discussion

### 4.1. Correlation between Phylogroups and Proteomes as Revealed by SELDI-TOF Mass Spectrometry

Intraspecies diversity of a microbial population as reflected in protein patterns has long been assessed by methods such as SDS-PAGE or isoelectric focusing and shown to correlate with a high degree of confidence with genomic methods such as DNA-DNA hybridisation. However, at the intraspecies level, more recent genomic-based methods such as MLST yield higher-resolution typing compared to recent protein profiling methods such as MALDI-TOF mass spectrometry [20, 21] and rarely do strain types correlate with both methods. It is our view that standard MALDI-TOF-MS-based approaches of either whole cells or formic acid extracts of cells provide excellent identification of a strain to the species level and now supersede the resolution of 16S rDNA. However, typing methods based on MALDI-TOF-MS which produce most of its mass ions in the range 1–10 kDa provide insufficient mass peaks for typing and data derived from such studies are often based on a few mass ions and are often subjective. However, in *P. acnes, *MALDI-TOF-MS has the capacity to differentiate different phylogroups suggesting that they may comprise the nucleus of new taxa (unpublished work, [22]). Because of the nature of selective capture of the ProteinChip arrays, SELDI-TOF-MS analyses specific classes of proteins and the mass range extends beyond standard MALDI-TOF-MS. In this study, the mass range for *P. acnes* using the Q10 ProteinChip array was extended up to 35 kDa and categorised strains into four distinct patterns. These correlated with three types of one atypical strain, K56 (type IA_2_). Furthermore, the profiles of each group were consistent for cells under anaerobic and aerobic conditions. Of the four groups, A was the most complex and encompassed strains from phylogroups IA_1_, IA_2_, and IB. Group B comprised strains solely from type II whereas Group C strains belonged to type III. The remaining Group D comprised types IA_2_ and II. 

Proteomic spectra for strains from phylogroups IA_1_, IA_2_, and IB showed very similar patterns and these could not be distinguished. This suggests these groups express a very similar proteome and may colonise a common niche. This result is consistent with the report by Brzuszkiewicz et al. [23], in which they showed the gene expression pattern in strain 266 (type IA_1_) and strain KPA171202 (type IB) using an RNA microarray system. Their analysis demonstrated some differences between these strains, but it was relatively small, within a 30% range of the transcriptome representing each gene. The present data also concurs with the work by Holland et al. [24], who showed a profile of secreted proteins of five strains that represent four lineages, IA, IB, II, and III, using 2D gel electrophoresis and MALDI-MS identification. Their results suggested that intratype I difference of secreted proteins between three stains of subtypes IA and IB is much smaller than intertype differences. While the present data is not limited to secreted proteins, the overall findings between these studies are consistent. Holland et al. studied the expressed proteome of *P. acnes* of cell grown to mid-log phase in a liquid BHI broth and reported a number of interesting features on the secreted protein. The work undertaken here had a different focus, that is, to develop a proteome-based typing system to probe the microhabitat of various strains eventually. Cell profiles were compared at a selected time of 5 days when mass spectra were stable and robust. The method used here also focused on analysing the most abundant proteins as a starting point for more detailed analysis using artificial neural networks.

Groups C and D were heterogeneous. Group C consisted of four strains of type II and four strains of type I. Interestingly, these four type I strains moved from Group A to Group C when cultured aerobically. Group D consisted of five strains of type II and one atypical strain of type IA_2_. This atypical strain (K56) repeatedly showed the same spectral pattern. The explanation for the recovery of this strain in this group is uncertain and may become clearer as more strains are analysed.

### 4.2. Difference of Proteomes between Anaerobic and Aerobic Culture Conditions

While the genome provides the blueprint of a cell, the proteome reflects its expression and responds to changes in its habitat; regulating different proteins to survive and proliferate in its new environment. Mass spectrometry is a useful tool to gain insight into changes in the proteome. SELDI-TOF mass spectrometry was used in the first instance here to assess its potential to detect partial changes in the proteome of cells grown in aerobic and anaerobic environments. Ideally, the combined spectra of mass ions derived from a range of ProteinChip arrays should be used to gain a more holistic overview; however, this was not possible and a single ProteinChip, Q10, that yielded the most dense and reproducible mass ions spectra was selected. There was unambiguous evidence of differences of the overall spectra of cells grown under the two culture conditions, and each change seemed unique to the spectral groups. Results are represented as a heat map in which both similarity of mass ions and mass intensity were compared for all strains. To further analyse these complex datasets, a stepwise ANN modelling approach was used to dissect a combined panel of mass ions from the SELDI-TOF spectra that relate to the changes seen between aerobic and anaerobic samples and these are given in Table 2. The ANN analysis clearly pointed out three characteristic peaks, and this result supported the idea that some universal differences exist between these two culture conditions. 

High-resolution CGE analysis was carried out on one strain, K72, an IB type to reinforce the observation of differences of charged proteins between the two culture conditions that were revealed by SELDI-MS. The DeltaDOT system explored here for the first time is a new type of rapid, high-resolution protein separation technology using capillary electrophoresis and while gross changes were clearly evident between cells grown aerobically against one grown anaerobically (Figure 3), a drawback of this approach at present is that the technology does not have a means to tap off peaks of interest for MS/MS analysis. It is anticipated that in the future this will be possible and would provide a new, rapid, high-throughput means of undertaking such analysis.

Consequently, we carried out LC-MS/MS using an Orbitrap Mass Spectrometer (ThermoFisher Scientific) to analyse which proteins contribute to changes in expression profiles when cells are grown under aerobic and anaerobic conditions. 1D-SDS-PAGE profiles for cells grown under microaerophilic and anaerobic conditions were similar but different from cells grown aerobically. There was a significant visible increase in protein expression between microaerophilic/anaerobic growth to the corresponding segment of the gel for cells grown aerobically (molecular weight 12–15 kDa; see Figure 4). Detailed protein analysis of this portion of the gel was undertaken by removing this section of the gel, digesting the proteins with trypsin, and subjecting them to LC-MS/MS. A total of 19 proteins were identified (see the previous list of proteins). 

Proteins that were significantly upregulated under anaerobic and microaerophilic conditions belong to a broad functional categories. For example, phosphoenolpyruvate-dependent sugar phosphotransferase system EIIA 2 is a protein involved in the regulation of a variety of metabolic and transcriptional processes. Membrane-anchored proteins, antioxidant AhpC/TSA family, have antioxidant activity that could remove peroxides or H_2_O_2_. Several ribosomal proteins involved in processing genetic information were also identified. Another interesting protein identified and upregulated under anaerobic and microaerophilic conditions is methylmalonyl-CoA epimerase, a protein involved in the propionate-producing methylmalonyl-CoA pathway that produces short-chain fatty acids, such as propionic acid [25]. Propionic acid is a key metabolic end product of this species and is even reflected in its nomenclature. 

Of particular interest, the Christie-Atkins-Munch-Petersen (CAMP) factor, a cohaemolytic protein that has been characterised as a pathogenic determinant exerting lethal effects when administered to laboratory animals [23], was identified upregulated under anaerobic and microaerophilic conditions. In our analysis of the complete and draft genome sequences of *P. acnes* strain KPA171202 (type IB), five genes (PPA687, PPA1198, PPA1231, PPA1340, and PPA2108) are encoded in the genome as homologs to CAMP factors [24]. Using a peptide AFAPANVLNIIGK as a query, BLASTp against nonredundant NCBI protein sequences (nr) database, the identified protein was assigned to the gene nomenclature of the *P. acnes* KPA171202 genome as PPA1340 (CAMP1, accession: YP_056047); a CAMP factor is both secreted and surface-associated that was preferentially expressed in the exponential growth phase [23]. The measurement of its virulence is difficult to assess using animal models or *in vitro* cell lines as these are not the natural habitat of the species and only provide clues to such mechanisms. However, it is generally considered that type IB isolates are nonpathogenic; consequently the finding of at least one CAMP factor in K115 (type IB) grown in an anoxic environment is interesting. It was suggested that *P. acnes* CAMP factor may hijack host acid sphingomyelinase to degrade and invade host cells [26]. For *P. acnes *to invade the host cells, it would need to switch to an anaerobic metabolism as the redox potential is likely to decrease significantly from the surface of the skin to deeper tissues. The question as to whether anaerobic isolates are more successful pathogens or whether the *E*_*h*_ of the local environment is the basis for selective pressure and success of strains of *P. acnes *as a pathogen remains open. 

Current work is in progress to analyse the complete proteome directly by MS/MS since many of the changes in the proteome will not be readily apparent due to differences in staining or may be in low abundance. These analyses will also encompass a larger number of strains from various phylogroups grown under these conditions to help elucidate the specificity of the functional changes that accompany these imposed environmental parameters.

## 5. Conclusion

The present study demonstrated a good correlation between genomic and proteomic analyses and showed that each subtype expresses a different proteomic profile, and these were categorised into four main proteomic groups. The intracellular protein expressions differ in anaerobic/microaerophilic and aerobic culture conditions, and the differences were unique among each spectral group. Further studies are in progress to characterize some of these biomarkers to shed light on the basis for their selection.

## Figures and Tables

**Table 1:** Phylogroup profiles of *P. acnes* strains used in this study.

Type	MLST ST*	Strain name
IA_1_	6	K107
IA_2_	9	K56
	22	K51, K72
IB	10	K12, K81, K86, K114, K115
II	42	S13, S14
	43	K15, K79, K80, K106, K127
	46	K96
III	19	K55, K78, K124

*MLST sequence types following the scheme by McDowell et al. [11].

**Table 2:** Universal peak differences between anaerobic and aerobic samples as revealed by stepwise calculation using Artificial Neural Network analysis.

Peak difference	Higher peak samples
(approx. m/s)	(Anaerobic or aerobic)
9493	Aerobic
13492	Anaerobic
19134	Aerobic
